# Long-Term Vision and Non-Vision Dominant Behavioral Deficits in the 2-VO Rats Are Accompanied by Time and Regional Glial Activation in the White Matter

**DOI:** 10.1371/journal.pone.0101120

**Published:** 2014-06-26

**Authors:** Xue Song Tian, Xian Jun Guo, Zhi Ruan, Yun Lei, Yu Ting Chen, Hai Yan Zhang

**Affiliations:** 1 CAS Key Laboratory of Receptor Research, Shanghai Institute of Materia Medica, Chinese Academy of Sciences, Shanghai, China; 2 Department of Pharmacology, Shanghai Institute of Materia Medica, Chinese Academy of Sciences, Shanghai, China; University of Modena and Reggio Emilia, Italy

## Abstract

The permanent occlusion of common carotid arteries (2-VO) in rats has been shown to induce progressive and long-lasting deficits in cognitive performance, however, whether these aberrant behaviors are attributed to visual dysfunction or cognitive impairment and what are the underlying mechanisms, remain controversial. In the present study, vision dominant (Morris water maze) and non-vision dominant (voice-cued fear conditioning) behavioral tests were assigned to comprehensively evaluate the influence of 2-VO lesion on cognitive behaviors. In the Morris water maze test, escape latencies of 2-VO rats were markedly increased in both hidden and unfixed visible platform tasks, which were accompanied by severe retinal damage. In the voice-cued fear conditioning test, significant reduction in the percentage of freezing behavior was observed at 60 days after 2-VO lesion. Chronic lesion by 2-VO failed to cause noticeable changes in the grey matter, as indicated by intact hippocampal and prefrontal cortical structures, sustained synaptic protein levels and glial cell numbers. In contrast, aberrant arrangement of myelinated axons was observed in the optic tract, but not in the corpus callosum and inner capsule of 2-VO rats. Concurrently, marked astrocyte proliferation and microglia activation in the optic tract occurred at 3 days after 2-VO lesion, and continued for up to 60 days. Differently, robust glial activation was observed in the corpus callosum at 3 days after 2-VO surgery, and then gradually returned to the baseline level at 14 and 60 days. Our study reported for the first time about the effect of 2-VO on the long-term cognitive impairment in the non-vision dominant fear conditioning test, which may be more applicable than the Morris water maze test for assessing 2-VO associated cognitive function. The time and region specific glial activation in the white matter may relate to retinal impairment, even behavioral deficits, in the setting of chronic cerebral hypoperfusion.

## Introduction

Vascular dementia (VaD), as the second most common form of dementia in the elderly, accounts for approximately 20% of dementia cases [1]. It is well known that a decrease in cerebral blood flow (CBF) precedes the onset of VaD [2], and close coupling was evident between reduced CBF and cognitive dysfunction in patients with dementia [3]. Based on the fact that rats have complete circle of Willis which can affords constant blood flow after the onset of occlusion, permanent bilateral common carotid artery occlusion (two-vessel occlusion; 2-VO) in rats has emerged as a suitable approach to unravel the effect of chronic cerebral hypoperfusion on cognitive dysfunction and neurodegenerative processes [4], [5], and evaluate the efficacy of drug candidates for the treatment of VaD [6], [7].

Although accumulating studies have indicated that 2-VO treatment leads to impaired learning and memory behaviors in rats [5], [7], [8], this conclusion remains questionable due to varied behavioral performances [9]–[11] and 2-VO associated vision problems [12]–[14] in different strains of rats. It is well-known that Morris water maze (MWM) is one of the most frequently used tests to assess spatial learning and memory [15], [16] in rats, by judging their ability to find a hidden, submerged target platform that cannot be seen, heard or smelt in a circular pool of water [17]–[19]. However, as the accomplishment of the task depends largely on the integrity of vision system, 2-VO-associated vision abnormalities will obviously impact the accuracy of cognitive performance assessment. It is still lack of reports about long-term effects of 2-VO on cognitive behaviors although short-term efficacy is well studied [20], [21]. Therefore, it is crucial to evaluate the long-term effect of 2-VO lesion on both vision and non-vision associated cognitive performances employing proper behavioral tests.

Over the years, inflammatory responses have been increasingly demonstrated to play a detrimental role in the pathogenesis of chronic ischemic injury [5], [22], [23]. However, there is still a lack of evidence about the dynamical alternations of inflammatory responses in both white matter and grey matter during the pathological process of 2-VO lesion. Therefore, the present study was designed to 1) observe the cognitive performances on both vision and non-vision dominant behavioral tests at 2 months after 2-VO lesion; 2) simultaneously evaluate morphological changes of the retinal, white matter and grey matter structures at 2 months after 2-VO lesion; 3) assess the time-related changes of inflammatory responses in the grey matter and three regions of white matter, at 3, 14 and 60 days after 2-VO lesion. Our previous data and recent reports [12], [24], [25] suggest that almost all of Wistar rats lost pupillary light reflex (PLR) after 2-VO surgery, while only about half of Sprague-Dawley (SD) rats lost PLR. Therefore, Wistar rats were used in the current study in order to reduce the inconsistent influence of 2-VO lesion on vision system.

## Materials and Methods

### Ethics Statement

All the animal experiment protocols were approved by the Institutional Animal Care and Use Committee of Shanghai Institute of Materia Medica. All the rats were maintained in the specific pathogen-free and Association for Assessment and Accreditation of Laboratory Animal Care International (AAALAC) approved animal facility.

### Animals and reagents

Fifty male Wistar rats (280–300 g) were obtained from shanghai laboratory animal center, Chinese Academy of Sciences. Four rats were kept in a cage and allowed free access to water and food. The house was maintained at 22–25°C with a 12-hr light-dark cycle.

Mouse monoclonal anti-PSD-95 (postsynaptic density protein-95) and anti-synaptophysin were respectively obtained from Santa Cruz Biotechnology (sc-32290) and Abcam Inc (ab8049). Rabbit polyclonal anti-IBA (ionized calcium binding adapter) was purchased from Wako (019-19741). Mouse monoclonal anti-GFAP (Glial fibrillary acidic protein) was purchased from EMD Millipore (MAB3402). Anti-mouse IgG, HRP conjugate was purchased from Kang Chen Biotechnology (KC-MM-035). Anti-mouse IgG-FITC and anti-rabbit IgG-Rhodamine were purchased from Jackson Biotechnology (115-095-003, 111-025-003).

### 2-VO surgery

2-VO surgery was performed as previously described [6]. Briefly, rats were anesthetized with pentobarbital (50 mg/kg, i.p.), and bilateral common carotid arteries were gently separated from the carotid sheath and vagal nerves. Each artery was bi-ligated with 6–0 silk sutures, and then the artery was cut between the two ligatures. The body temperature of rat was maintained at at 37±0.5°C with a heating pad throughout the surgical procedure. Sham-operated controls received the same operation without ligation.

### Behavioral assessment

**Figure 1:**
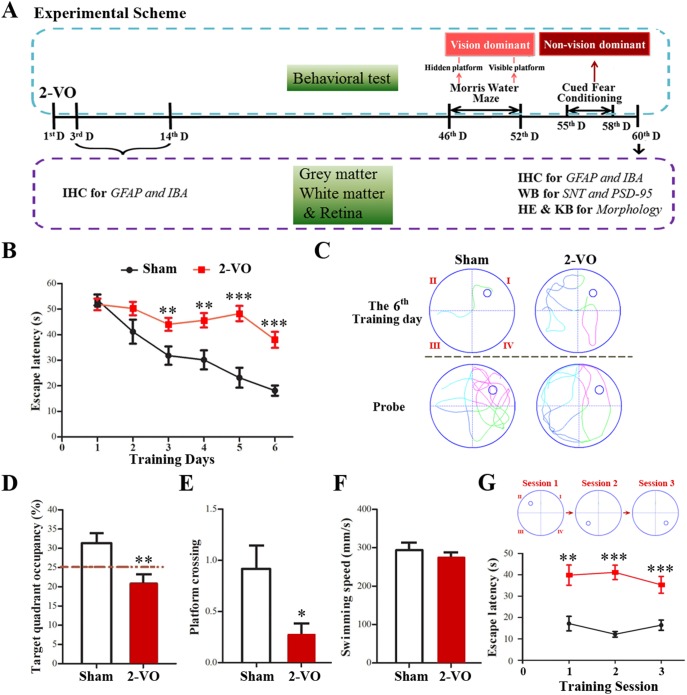
Bilateral common carotid artery occlusion induced impaired cognitive performances on the hidden platform and visible platform tasks of MWM. (A) Experimental design. (B) The escape latency of rats in the training trials of hidden platform task. The 2-VO group showed significantly longer escape latencies than that of the sham group. (C) Representative pathways in the last training day and probe trial. (D) Percentage of time spent in the target quadrant in the probe trial showing memory impairment of the 2-VO rats. (E) Frequency of platform crossing in the probe trail. The 2-VO group showed significantly reduced frequency than that of the sham group. (F) Swimming speed in the probe trail. There is no difference between the 2-VO and sham group. (G) The escape latency of rats in the unfixed visible platform task. The 2-VO group showed significantly longer escape latencies than that of sham group. Values are expressed as mean ± SE; Sham group: n = 12; 2-VO group: n = 16; *P<0.05, **P<0.01, ***P<0.001 vs. sham group. Abbreviations: D, day’ IHC, immunohistochemistry; IBA, ionized calcium binding adapter; GFAP, glial fibrillary acidic protein; PSD-95, postsynaptic density protein-95; SNT, synaptophysin.

To study cognitive behaviors of rats as well as the impact of vision on these behaviors, vision dominant hidden and visible platform tasks of MWM and non-vision dominant voice-cued fear conditioning tests were performed at 46 and 55 days after 2-VO occlusion, respectively (Figure 1A).

### Morris water maze performance (hidden platform task)

The maze was consisted of a circular tank (170 cm in diameter, 60 cm high) and was divided into four quadrants. It was filled with water (35 cm in height) and the water temperature was maintained at 21–22°C and made opaque by Chinese ink. A black platform (10 cm in diameter) was located in the center of quadrant I (1.5 cm below the water surface). Prominent external cues attached on the curtain surrounding the tank were used to indicate the location of the platform. The rats were subjected to the MWM using a procedure as described previously [6]. Rats were given three trials per day for 6 consecutive days to locate the hidden platform with an inter-trial interval of 12 min. In each trial, rats were released into the water facing the wall of the pool at a novel starting point. The latency to the hidden platform was recorded. If the rats could not find the platform within 60 s, they were gently guided to the platform and allowed to remain on it for additional 20 s, and then 60 s was recorded as the escape latency. Swimming path of the rats was monitored by a video camera linked to a video recorder and tracking device (DigBehv-MM, Jiliang Software Technology, Shanghai, China). A probe test was conducted on the 7^th^ day. Briefly, the platform was removed, and the rats were released from the starting point most far away from the platform and allowed to swim freely in the pool for 30 s. The time spent in the target quadrant, the frequency of platform crossing over the original platform, and the swimming velocity were recorded.

### Morris water maze performance (unfixed visible platform task)

Visible platform test was performed after completion of the probe test, with a white platform protruded 1 cm above the surface of the water located in quadrant II. Each rat was given 2 trials to locate the platform originated at two different starting points, with a maximum period of 60 s for each trial, and allowed to remain on the platform for additional 20 s. The escape latency was recorded. The same tests were then repeated with the visible platform translocated to quadrant III and quadrant IV sequentially.

### Voice-cued fear conditioning performance (non-vision dominant)

Similar as described previously [26], [27], the non-vision dominant cued fear conditioning task was composed of two parts: training session and cued test session. In the training session, the rats were allowed to explore the chamber (25 cm×31 cm×25 cm, Med Associates) freely for 3 min to get familiar with the context. After the habituation, conditioned stimulus (CS) with a 75 decibel (dB) tone with an intensity of 5000 HZ was continued for 6 s, followed by unconditioned stimulus (US) with a 0.3 mA foot shock lasting for 1 s. The CS-US pairings were repeated 6 more times with an inter-stimulus interval of 30 s. Sixty seconds after the last shock, the rats were returned to their home cages. The cued test session was conducted 72 h later. To create a distinct context, two side walls and the ceiling were changed by adding a plastic curved white plate and the floor was changed to a white plastic plate. The rats were placed in the novel context and allowed to explore it freely for 3 min. Seven times of CS were then given with a 30 s interval. Thirty seconds after the last CS, the rats were returned to their home cages. The chambers were cleaned with 1% acetic acid after each trial to eliminate smell. Freezing was defined as no visible movement except for breathing. All events were recorded and analyzed by Med Associates software.

### Tissue preparation

For histological morphology and immunohistochemistry study, animals were deeply anesthetized with pentobarbital (80 mg/kg, i.p.) and perfused transcardially with 0.9% saline followed by 4% paraformaldehyde in 0.1 M phosphate buffer (pH 7.4). Brains and eyes were immediately collected and placed in 4% paraformaldehyde for post fixation. Subsequently, tissues were embedded in paraffin and cut into sections of 4 µm thickness for hematoxylin and eosin (H&E) staining and immunohistochemistry study or 8 µm for Klüver-Barrer (KB) staining using a Micro-Tom (HM 340E, Thermo Scientific, Walldorf, Germany). For western blotting analysis, hippocampus and prefrontal cortex were dissected on ice immediately after rats were decapitated.

### Histological morphology and immunofluorescence

To study the histological morphology and immunohistochemistry, eyes and brains of rats were collected at 3, 14 or 60 days after 2-VO surgery (Figure 1A). Sections were deparaffinized in xylene and rehydrated in a graded series of ethanol. For morphological examination, the deparaffinized sections were stained with H&E and photographs were taken with a digital camera. To perform immunohistochemistry, the sections were undergone antigen repairment using microwave in citrate buffer and then blocked with 5% bovine serum albumin (BSA) and 5% horse serum in 0.01 M phosphate buffered saline (PBS) for 30 min at room temperature. Subsequently, the sections were incubated with primary antibodies against GFAP (1∶500) and IBA (1∶200) overnight at 4°C, followed by incubation with corresponding secondary antibodies for 1 h at room temperature. After cover-slipped, the sections were visualized and images were captured using Olympus IX-81 Confocal microscope.

### Klüver-Barrer staining

Sections were deparaffinized and hydrated with 95% ethanol, and then stained with 0.1% luxol fast blue solution at 56°C overnight. The sections were differentiated in 0.05% lithium carbonate solution and 70% ethanol until gray matter clear and white matter sharply defined. After dehydration in ethanol and transparency in xylene, the sections were cover-slipped and visualized under light microscope. The severity of the white matter changes was graded as normal (grade 0), disarrangement of the nerve fibers (grade 1), formation of marked vacuoles (grade 2), and disappearance of myelinated fibers (grade 3) [28], [29].

### Western blot analysis

The procedure of western blot was similar as described previously [30]. Rats were sacrificed after MWM. The hippocampus and prefrontal cortex were quickly collected on ice, frozen immediately in liquid nitrogen, and kept at –70°C until used. Brain tissues were then homogenized in RIPA buffer, and centrifuged at 12,000 g for 10 min at 4°C. Protein concentration was determined by Bio-Rad protein assay (Bio-Rad, Hercules, CA). Tissue homogenates (40 µg protein) from the hippocampus and prefrontal cortex of each rat were boiled in sodium dodecyl sulfate (SDS) sample buffer for 5 min, electrophoresed on 10% SDS-polyacrylamide gel, and transferred to the polyvinyl difluoride membrane (Bio-Rad). Membranes were blocked with 5% nonfat dry milk in Tris-buffered saline containing Tween-20 (TBS-T, 2 mmol/L Tris-HCl, 50 mmol/L NaCl, 0.1% Tween 20, pH 7.5) for 1 h at room temperature and subsequently incubated with primary antibodies for synaptophysin and PSD-95 (1:2000) overnight at 4°C. The membranes were then washed three times with TBS-T containing 0.1% Tween 20, and treated with horseradish peroxidase-conjugated anti-mouse IgG (1∶10,000) for 1 h at room temperature. The signal was detected using enhanced chemiluminscent (ECL) reagent (34080, Thermo Scientific, Rockford, USA) and analysis software (Image J, Broken Symmetry Software, Bethesda, MD, USA).

### Statistical analysis

The morphological and behavioral assessments were performed by treatment-blinded investigators. All of the data were expressed as mean ± SE. Results of MWM and cued fear conditioning test were first statistically analyzed by repeated measures (General Linear Model), and then individual day comparisons were analyzed by Student’s t-test, which was also used to analyze results from time spent in the target quadrant and synaptic proteins expression. GFAP and IBA immunohistochemistry were analyzed by ANOVA followed by post hoc test of Least Significant Difference (LSD) to identify significant differences. The frequency of platform crossing, average percentage of freezing and the severity of the white matter demyelination between the groups were analyzed by the non-parametric Mann-Whitney U test. *P* values < 0.05 were considered statistically significant.

## Results

### Bilateral common carotid artery occlusion induced vision dominant and non-vision dominant cognitive performance deficits

Cognitive performance deficits caused by 2-VO were assessed in the MWM test that are based on visual cues. The escape latencies to find the hidden platform in all Wistar rats during the acquisition session are shown in Figure 1B. During the training, rats in the 2-VO group exhibited longer escape latency. Comparisons of individual day values are as follows: at the first training day, the sham and 2-VO groups both took more than 50 s to find the hidden platform. Escape latency was relatively stable in the 2-VO group on the following days, whereas it gradually decreased in the sham-operated groups. Furthermore, the 2-VO operated rats began to have notably longer escape latencies than sham-operated rats at the third training day (*P*<0.01), which lasted for 4 days (*P*<0.01 at the fourth day and *P*<0.001 thereafter). The typical swimming path of the last training day indicates that the 2-VO rats may use an inappropriate searching strategy to locate the hidden platform, which resulted in longer latency (Figure 1C; upper panel).

Representative examples of typical swimming path in the probe trial indicate that 2-VO lesion decreases the frequency of platform crossing as well as the time spent in the target section (Figure 1C; lower panel). In the probe trial, swimming time in the target quadrant was used to evaluate the retention performance. The sham-operated group swam longer in the target quadrant than the 2-VO group (*P*<0.01, Figure 1D). The 2-VO rats spent more time in the border of pool, while less time in center of the pool than the sham-operated group (data not shown). In agreement, frequency of platform crossing was decreased in the 2-VO group (*P*<0.01, Figure 1E). No significant difference was observed between rats in sham group and 2-VO group in the swimming velocity (Figure 1F).

In order to evaluate the influence of visual impairment on the cognitive performance dysfunction in the 2-VO rats, we tested the ability of rats to locate a visible platform in the MWM. In this vision dominant behavioral test, and found a significant difference between the two groups (*P*<0.01, Figure 1G): the sham-operated rats reached the platform in less than 20 s, while the 2-VO rats took more than 40 s, which indicating that the impaired spatial learning and memory performances of the 2-VO rats may be attributed partly to visual problem.

### Bilateral common carotid artery occlusion induced retinal degeneration

**Figure 2:**
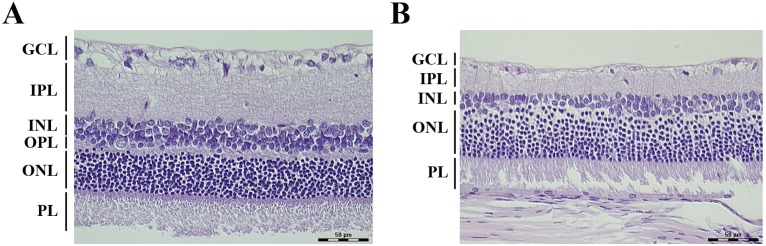
Bilateral common carotid artery occlusion induced degeneration in the retina of rats. All images were taken in the peripheral retina (3 mm from the optic disc). (A) Sham group. (B) 2-VO group (at 2 months after 2-VO surgery). 2-VO caused severe overall retinal degeneration. Scale bar: 50 µm. Sham group: n = 5; 2-VO group: n = 6. Abbreviations: GCL, ganglion cell layer; IPL, inner plexiform layer; INL, inner nuclear layer; OPL, outer plexiform layer; ONL, outer nuclear layer; PL, photoreceptor layer.

The 2-VO rat model has also been applied for ischemic eye research. Histological examinations with H&E staining showed that significant damage was distributed along the entire layers of the retina of rats subjected to 2-VO (Figure 2B), compared with that of the sham group (Figure 2A). After 2 months of 2-VO lesion, the numbers of large cells (presumed to be retinal ganglion cells) were reduced in the ganglion cell layer (GCL), and the thickness of the inner plexiform layer (IPL) was decreased (Figure 2B). In the inner nuclear layer (INL), cell loss and vacuolation became manifest, and the band in the outer plexiform layer (OPL) disappeared (Figure 2B). The nuclei of photoreceptors in the outer nuclear layer (ONL) were relatively preserved after 2 months of 2-VO lesion (Figure 2B).

### Bilateral common carotid artery occlusion induced decreased freezing in non-vision dominant cued fear conditioning test

**Figure 3:**
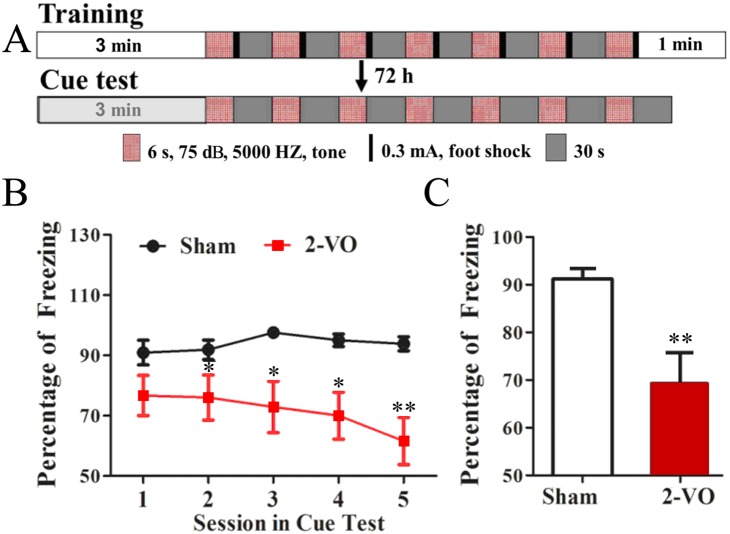
Bilateral common carotid artery occlusion decreased percentage of freezing behavior in a non-vision dominant cued fear conditioning test. (A) The schematic of voice-cued fear conditioning test. (B) The percentage of freezing of the sham and 2-VO groups in the first five sessions. (C) The average percentage of freezing of the sham and 2-VO groups in the first five sessions. Sham group: n = 12; 2-VO group: n = 16; *P<0.05, **P<0.01 vs. sham group. Abbreviations: s, second;dB, decibel.

As above-mentioned, vision was involved in the MWM test. In order to better evaluate the cognitive function, a non-vision dominant cued fear conditioning test was employed consequently. Figure 3A shows procedure of the behavioral test. After experienced seven pairs of tone-shock, rat exhibited freezing behavior even when only tone was present in the cued test, which means they have remembered the association between tone and shock. In the cued test, freezing percentages of the 2-VO rats gradually decreased in the first five sessions and were significantly lower than that of sham group since the second session (*P*<0.05 or *P*<0.01, Figure 3B). As a result, the average freezing percentage of the 2-VO rats in the first five sessions was significantly lower compared with that of the sham rats (Figure 3C). The results of non-vision dominant cued fear conditioning test indicate that cognitive function might be impaired by 2-VO lesion.

### Bilateral common carotid artery occlusion did not induce evident changes in morphology, synaptic proteins expression and glial cells number in the hippocampus and prefrontal cortex

**Figure 4:**
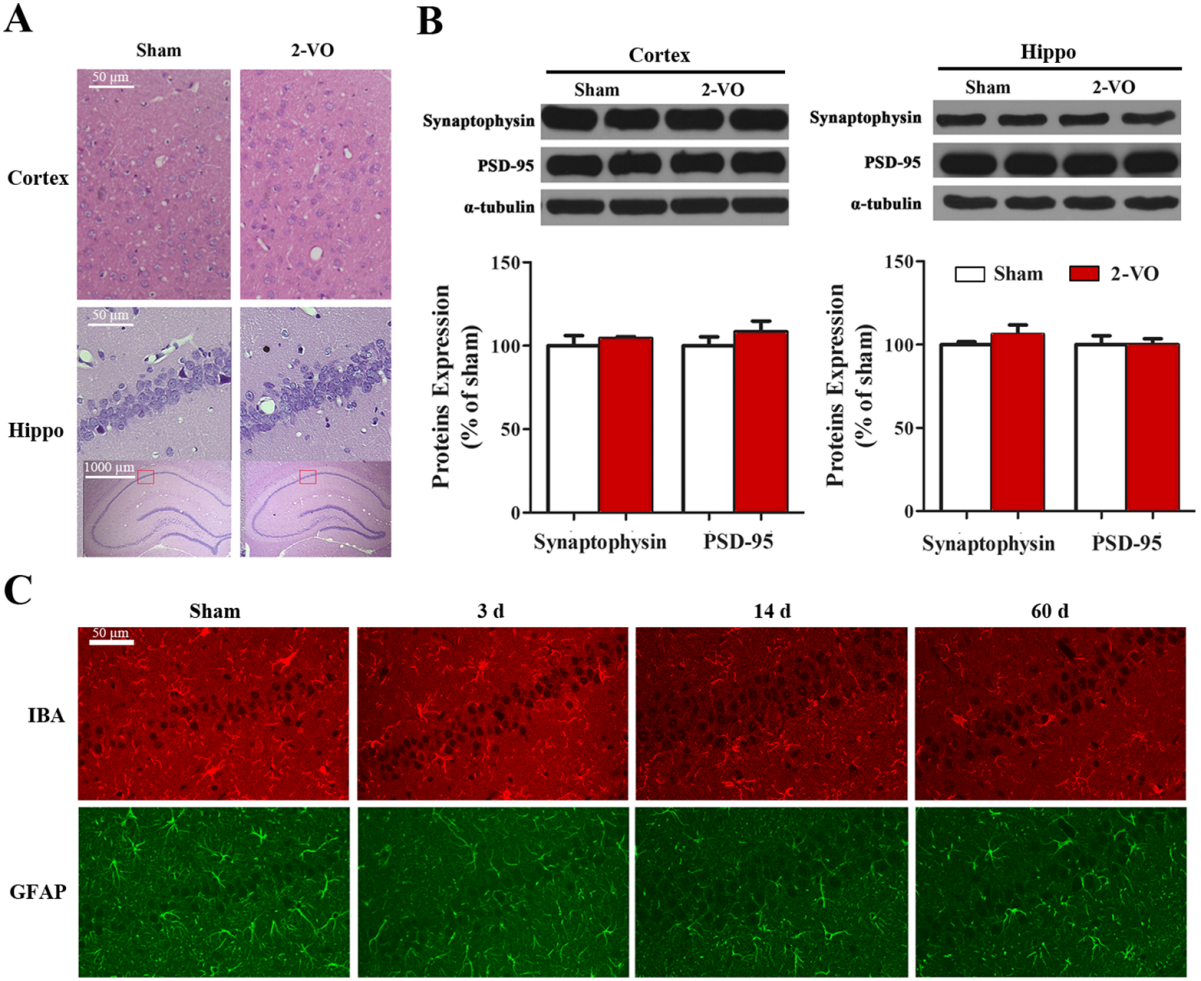
Preservation of histological morphology, synaptic proteins expression and glia cells number in the prefrontal cortex or hippocampus of rats subject to bilateral common carotid artery occlusion. (A) Representative H & E staining images of the prefrontal cortex and hippocampus. (B) Representative western blot bands and protein expression percentages of synaptophysin and PSD-95. (C) Representative IHC images of IBA and GFAP in hippocampus. Scale bar: 50 or 1000 µm. Sham group: n = 5; 2VO group: n = 6. Abbreviations: IHC, immunohistochemistry; IBA, ionized calcium binding adapter; GFAP, glial fibrillary acidic protein; PSD-95, postsynaptic density protein-95.

Prefrontal cortex and hippocampus are closely associated with cognitive function and reported to be vulnerable to ischemia. To detect whether the cognitive dysfunction in voice-cued fear conditioning test was due to prefrontal cortical or hippocampal damage, their morphology, synaptic proteins expression and number of glia cells were assessed. Figure 4A showed that there was no evident morphological abnormality in the Wistar rats at 60 days after 2-VO surgery as compared with the sham group. Moreover, the protein expressions of synaptophysin and PSD-95 were not different between two groups (Figure 4B), which indicates the synapses are not evidently affected by 2-VO lesion. Furthermore, the immunofluorescence intensities of IBA and GFAP in the hippocampus were not different between the 2-VO and sham groups at 3, 14 and 60 days after 2-VO lesion (Figure 4C), which demonstrated there was no obvious glial activation in the hippocampus.

### Bilateral common carotid artery occlusion induced white matter demyelination

**Figure 5:**
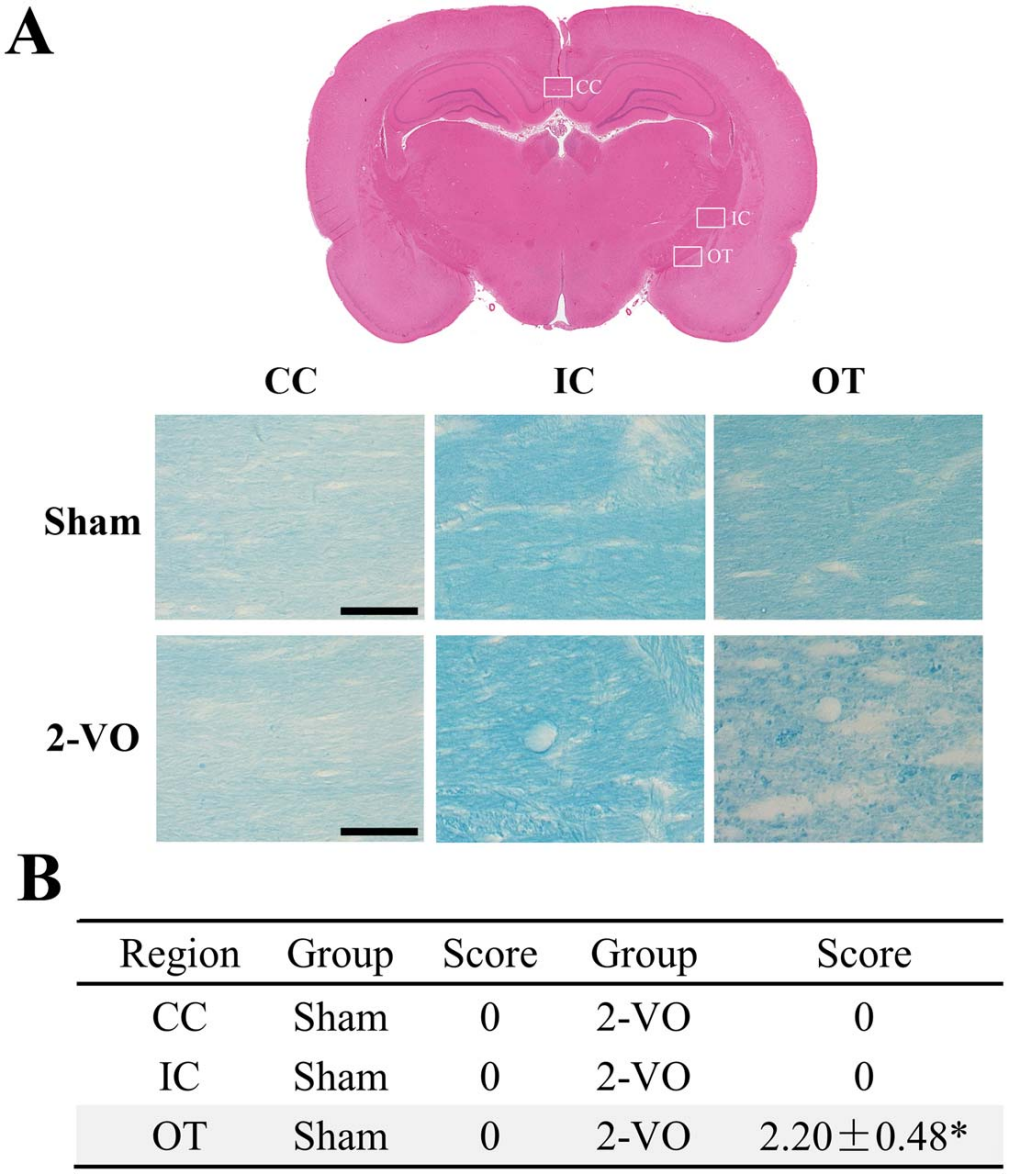
Bilateral common carotid artery occlusion induced white matter demyelination. (A) Representative KB staining images of the CC, IC and OT. (B) The severity of the myelinated axons changes in the CC, IC and OT. Scale bar: 50 µm. Sham: n = 5, 2-VO: n = 6. *P<0.05 vs. sham group. Abbreviations: CC, corpus callosum; IC, inner capsule; OT, optic tract.

White matter consists mostly of myelinated axons and transmits signals between different regions. It was reported that three regions of white matter, including corpus callosum (CC), inner capsule (IC) and optic tract (OT), were vulnerable to ischemia [31]. Therefore, these three regions of the white matter were studied by KB staining. Myelinated axons in the CC, IC and OT were arranged in an orderly fashion in the sham group (Figure 5A). However, marked vacuoles and disappearance of myelinated fibers in the OT region were observed at 60 days after 2-VO lesion, while the morphology in the CC and IC regions remained unchanged (Figure 5A). The degree of morphological damage was graded in the three regions by an independent observer who was blinded to the study. Results in table showed that the injury grades of the CC and IC were 0, which kept the same as the sham group (Figure 5B). Consistent with morphology results, the injury degree in the OT of the 2-VO group was 2.20±0.48, which was significantly higher than that of the sham group (Figure 5B).

### Bilateral common carotid artery occlusion induced glial activation in the white matter

**Figure 6:**
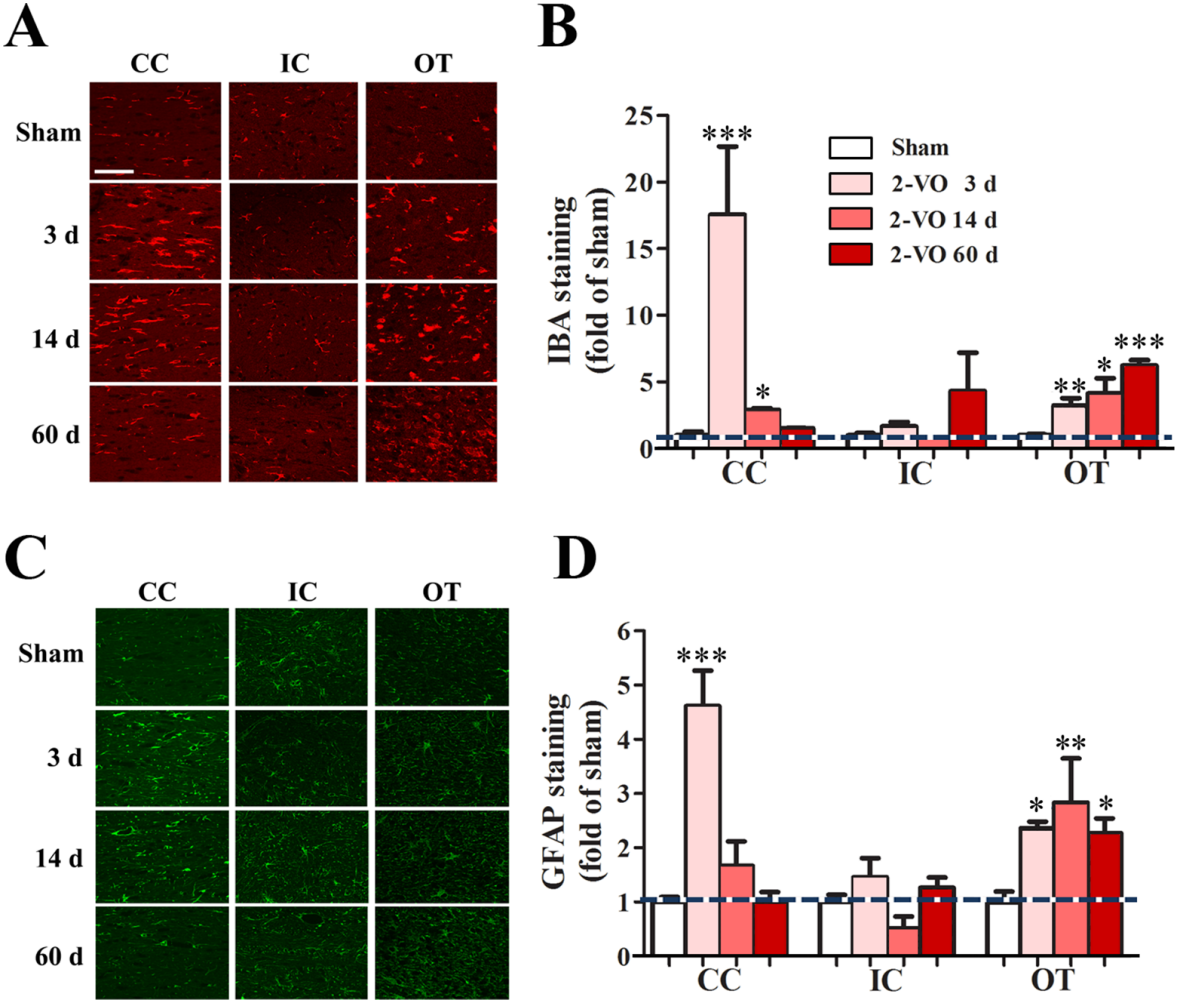
Bilateral common carotid artery occlusion induced overexpression of GFAP and IBA in the white matter of rats. (A) The representative IHC images of IBA. (B) Protein expression percentages of IBA. (C) The representative IHC images of GFAP. (D) Protein expression percentages of GFAP. Values are means ± SE, expressed as percentage of sham group which is set to 100%. Scale bar: 50 µm; Sham group: n = 5; 2-VO group: n = 6; *P<0.05, **P<0.01, ***P<0.001 vs. sham group. Abbreviations: CC, corpus callosum; IC, inner capsule; OT, optic tract; IHC, immunohistochemistry; IBA, ionized calcium binding adapter; GFAP, glial fibrillary acidic protein.

Microglia activation and astrocyte proliferation induce chronic inflammation, which might contribute to white matter impairment [5], [32]. Thus, astrocyte and microglia were detected by immunohistochemistry at 3, 14 and 60 days after 2-VO surgery. Microglia activation and astrocyte proliferation occurred in the CC and OT at all the three time periods, whereas they were not changed in the IC at any time spots we detected after 2-VO lesion (Figure 6). The proliferation intensity of IBA and GFAP in the CC reached peak at 3 days after 2-VO and then gradually dropped back, while the intensity of IBA and GFAP in the OT sustain increased at 3, 14 and 60 days after 2-VO lesion (Figure 6). The different changing patterns in the CC, IC and OT may reflect different vulnerability to ischemia. This result indicates that microglia activation and astrocyte proliferation might be related with the morphological white matter injury.

## Discussion

We have made a series of interesting observations in this work, i.e., 1) long-term cognitive performance deficits seen in the hidden platform task are likely to be associated with 2-VO induced visual impairment, which is confirmed by a new application of MWM using unfixed visible platforms and retinal structural damage; 2) for the first time reported the 2-VO induced long-term cognitive performance deficits in a non-vision dominant cued fear conditioning test designed to avoid the influence of visual system; 3) further extend the finding of “2-VO-induced white matter lesion in Wistar rats”, and suggest a robust but transient glial activation in the CC, a modest and sustained glial activation in the OT, and unchanged glial cell numbers in the IC after 2-VO injury. Our study indicates that 2-VO lesion has a long-term efficacy on both visual impairment and cognitive dysfunction in the Wistar rats, which may be relative to the retinal impairment and inflammatory responses in the white matter.

VaD can be studied using a 2-VO model that induces learning and memory impairments, white matter damage, retinal ischemia and neuropath logical changes [10], [33]. It is worth noting that experimenters must be careful to consider the role of visual sensory function in the performance of the MWM for 2-VO interrupt the retinal blood supply [34]. In consistent with previous reports [6], [16], [35], [36], we demonstrated a significant learning and memory impairment after 2-VO surgery in a 7-day hidden platform and probe trial learning and memory task (Figure 1B–F). This phenomenon may be closely associated with the longer escape latency of the 2-VO rats in the visible platform task (Figure 1G). We reset the location of the visible platform individually different for three sequential training sessions, in order to minimize the influence of non-vision associated factors on the behavioral performance. To further strengthen the finding about 2-VO induced visual impairment in behavior test, we investigated the effect of 2-VO lesion on retinal structure. As expected, severe structural damage in retina of 2-VO injured rats, which is in accordance with the previous studies that 2-VO could rapidly induce inner retina degeneration e.g. the thinner of GCL, IPL and INL, whereas the outer retina ONL was most resistant to the injury for blood supply difference in inner retina [14], [37], [38]. Taken together the poor performances of 2-VO rats in both hidden and visible platform tasks, it is obvious that the visual dysfunction may contribute to the impaired cognitive performance in the 2-VO rats.

To further clarify whether the cognitive function is injured by 2-VO lesion, we proceeded with a non-vision dominant behavioral assessment characterized by voice-cued fear conditioning test in the current study. In this measurement, a 75 dB tone with an intensity of 5000 HZ was used to induce the freezing performance, both 2-VO and sham rats were found to have normal reaction to the tone–can hear the voice and freeze after the tone stimulation. Moreover, a distinct chamber environment with different walls and ceiling was created on the day of test to eliminate the influence of visual cues. Therefore, under these specific experimental designs, the voice-cued fear conditioning test will be expected as a reliable choice to better reflect non-vision associated cognitive function in the 2-VO model. Interestingly, 2-VO rats still perform poorly in this behavioral task (Figure 3) with the avoidance of visual interference, which suggests that 2-VO lesion induced visual system impairment happened concurrently with the learning and memory deficit. Although previous study have indicated a poor learning ability in contextual fear conditioning test in the pregnant SD rats subject to 2-VO [39], our findings may provide more accurate evidence about the cognitive dysfunction, as previous study is conducted with SD rats–whose visual system is unstably affected by 2-VO, and contextual procedure–which requires functional visual system to fulfil the task.

Most of previous studies have attempted to relate the cognitive impairment to a loss in indices of white matter lesions (WMLs) or pyramidal cell death in the hippocampus [5], [40]–[42]. In our study, we demonstrate that above-mentioned cognitive performance deficiencies in both vision and non-vision dominant behavioral tests may not rely on severe cortical or hippocampal changes, at least morphological damage, because 2-VO lesion had no appreciable effect on the pathologic changes and the expressions of synaptophysin and PSD-95 in the prefrontal cortex and hippocampus (Figure 4A–C), suggesting that significant learning and memory impairment are not associated with the loss of synaptic contacts, and microglia/astrocyte proliferation in the grey matter of 2-VO rats. We also performed 2-VO surgery on another two cohorts of rats, and demonstrated that no significant morphological damage happened to the hippocampus of 2-VO rats using H&E-staining (data not shown). In agreement with our findings, many studies reported that 2-VO lesion cause no obvious focal damage [9], [25], [43], [44], or only transient abnormal messenger ribonucleic acid (mRNA) expressions in neurotropic factors [43], Alzheimer’s disease related factors [45] or brain lipid metabolism indexes [46] in the hippocampus of Wistar rats. However, there are also other studies reported a progressive neuronal cell death in the hippocampus of same rat strain subjected to 2-VO [47], [48], the discrepancy may due to the differences of genetic background of rat, gender or age of animals, or variations in the assay methodologies used.

Cerebral WMLs has received increasing attention as it has been found to coincide with cognitive and psychiatric disorders in the elderly and VaD patients. To be in agreement, the present study demonstrated that among the three white matter regions (CC, IC, OT) investigated currently, OT displayed the most striking degenerative changes, while the morphological staining of CC and IC remained relatively normal (Figure 5A–B). Therefore, in order to better understand the pathological process, we further systematically observed the inflammatory responses at various time points (both acute phase-3, 14 days and chronic phase-60 days) after the onset of 2-VO. The inflammatory responses in the white matter seems to be time-dependent and region-dependent, as the glial activation is robust but transient in the CC, while modest and sustained in the OT (Figure 6), which is in agreement with previous reports [5], [33], [49]. Activated microglia and astrocytes are major sources of pro-inflammatory factors (i.e. TNF-α, IL-1β), the overproduced inflammatory factors activate their corresponding receptors to initiate the intracellular apoptosis cascade, which may aggregate the WMLs, including vacuolation, rarefactions, axonal damage, and oligodendrocytes loss [50]. Our results further extend the previous findings that Wistar rats are more suitable for investigating WMLs [5], [25] instead of studying the grey matter injuries. Moreover, as is reported that lesion of OT might contribute to visual impairment, which could also be involved in the spatial learning deficit in the 2-VO rats [12], [51]. These results further indicate that 2-VO induced cognitive deficiency may be related with visual system injury and WMLs. In addition, many studies indicate that degeneration in white matter (especially CC) positively correlated with associative memory deficits [52] and cognitive impairment [53]. Therefore, it is still possible that the robust glial activation in the CC, although transient, may deteriorate the function of white matter and contribute to the later non-vision dominant behavioral deficits.

It is likely that 2-VO lesion is severe enough to damage the retina and in turn has a dramatic impact on the vision related cognitive impairment, however, 2-VO lesion induced time and region dependent inflammatory responses in the white matter are also evident, which may be associated with the vision and non-vision dominant behavioral deficits in the Wistar rats. Our results will shed more light on the understanding of cognitive and pathological alternations induced by experimental chronic hypoperfusion, and may provide useful clues for discovery of potential neuroprotective candidates for treating chronic cerebral ischemia associated diseases, including VaD.
